# The effect of female body mass index on cumulative live birth rate in women undergoing in vitro fertilization according to age

**DOI:** 10.1097/MD.0000000000037116

**Published:** 2024-01-26

**Authors:** Shuxia Ma, Ruirui Li, Lu Ouyang, Lin Li

**Affiliations:** a Reproductive Medical Center, Luoyang Maternal and Child Health Hospital, Luoyang, China.

**Keywords:** age, body mass index, cumulative live birth rate, female, in vitro fertilization

## Abstract

The aim of this study was to explore the impact of female body mass index (BMI) on cumulative live birth rates (CLBR) in patients treated with in vitro fertilization (IVF) and embryo transfer. A total of 2377 patients who visited the Reproductive Medical Center, Luoyang Maternal and Child Health Hospital from January 2015 to December 2021. The patients underwent the first IVF cycles. According to female BMI, patients were divided into 3 groups, group A: BMI ≤ 18.5 kg/m^2^ (underweight), group B: BMI: 18.5 to 24.0 kg/m^2^ (normal), group C: BMI ≥ 24.0 kg/m^2^ (overweight/obesity). Patient basic parameters and clinical outcomes were compared among these 3 groups. Multivariate logistic regression analysis was used to explore the impact of BMI on CLBR. In all treatment cycles, patients’ basic parameters were significantly different among 3 BMI groups. Age of underweight patient was younger than patients in the other 2 groups (28.45 ± 5.32 vs 29.89 ± 5.00 vs 30.74 ± 5.40; *P* = .000). In addition, number of oocytes retrieved was also significantly higher in group A (11.25 ± 5.97 vs 11.07 ± 5.49 vs 10.52 ± 5.02; *P* = .000). CLBR in these 3 groups were 66.40%, 65.98%, and 59.14%, respectively. In logistic analysis, overweight/obesity was associated with CLBR in young patients (aOR = 0.822, 95% CI: 0.817–0.957, *P* = .000). However, in the cycles of older patients, the effect of overweight/obesity on the CLBR was not significant (aOR = 0.986, 95% CI: 0.903–1.027, *P* > .05). Overweight/obesity is a predictor for CLBR in younger patients (<35 years old), but not in advanced age patients undergoing their first IVF/intracytoplasmic sperm injection treatment cycles.

## 1. Introduction

In recent years, with changes in social and living environments in particular, an increasing number of women have put more time and energy into educational and career development and have continued to postpone childbearing, causing the incidence of infertility to rise. Many infertile couples resort to in vitro fertilization (IVF) to achieve pregnancy, and IVF has become an effective means of treating infertility.^[1,2]^

With the development of the social economy and changes in living conditions, the number of overweight and obese people in the world has increased. Obese women of reproductive age have a significantly higher risk of infertility than normal-weight women.^[3]^ In the process of assisted reproductive treatment by IVF, patients with high body mass index (BMI) need higher doses of ovulation-inducing drugs and a longer duration of action. In addition, the influence of female BMI on oocyte quality, endometrial receptivity and the final clinical pregnancy rate has been a hot research topic in recent years.^[4,5]^ Although the conclusions of various studies are different, multiple studies have shown that overweight/obesity has an adverse effect on IVF pregnancy outcomes and increases maternal and fetal complications.^[6,7]^

To evaluate the final outcome of a patient assisted fertility cycle, it is necessary to consider both the fresh transfer cycle and the subsequent frozen-thawed embryo transfer (ET) cycle. The cumulative live birth rate (CLBR), as an evaluation indicator for a complete oocyte retrieval cycle, has more practical significance for patients than the previous clinical pregnancy rate and delivery rate.^[8]^ At present, studies are few and opinions inconsistent regarding whether female BMI affects the CLBR. Therefore, the purpose of this study was to investigate the effect of female prepregnancy BMI on the CLBR and to provide theoretical support for the clinical pretreatment of patients with high prepregnancy BMI.

## 2. Materials and methods

### 2.1. Research subjects

This study is a retrospective analysis of patients who visited the Reproductive Medicine Center of Luoyang Women and Children Hospital from January 2015 to December 2020 and underwent IVF/intracytoplasmic sperm injection (ICSI) for the first time. The follow-up time was 2 years from the start date of the ovarian stimulation cycle until December 31, 2022.

The inclusion criteria were as follows: first IVF/ICSI treatment cycles and achieved at least one live birth within 2 years from the start date of the ovarian stimulation cycle or all embryos have been used. The exclusion criteria were as follows: uterine malformations (e.g., unicornuate uterus, septate uterus); intrauterine adhesions and polyps; uterine fibroids and adenomyosis that seriously affect the shape of the uterine cavity; frozen sperm or oocyte freezing cycle; patients who did not achieve a live birth but still had remaining frozen embryos; or patients with incomplete data. This study was approved by the Medical Ethics Committee of Luoyang Women and Children Hospital. As data were deidentified and all analyses were retrospective, the requirement for informed consent was waived.

### 2.2. Research grouping and IVF/ICSI process

Patients were divided into 3 groups according to female BMI: Group A: BMI ≤ 18.5 kg/m^2^ (underweight), Group B: BMI: 18.5 to 24.0 kg/m^2^ (normal), and Group C: BMI ≥ 24.0 kg/m^2^ (overweight/obesity).

The CLBR was defined as the rate of first live births following the use of all fresh and frozen embryos derived from a single ovarian stimulation cycle (follow-up 2 years).

### 2.3. Ovarian stimulation protocols

The ovarian stimulation protocols included in this study included mainly GnRH agonist and antagonist protocol. Ovarian stimulation was started with a dose of 150 to 300 IU/d of follicle-stimulating hormone. According to the monitoring of follicle growth and serum hormone levels under transvaginal B-ultrasound, the dose of ovarian stimulation drugs was adjusted in real time. When 3 or more follicles ≥ 17 mm in diameter or 2 follicles ≥ 18 mm in diameter were detected by ultrasound, 5000 to 10,000 IU hCG was injected for triggering, and 34 to 37 hours later, oocytes were collected under transvaginal ultrasound guidance.

### 2.4. Fertilization method

Four to 6 hours after oocyte collection, the decision was made as to whether to perform IVF or ICSI according to the quality of the man semen.

### 2.5. Embryo quality assessment

The development of the embryo was recorded by embryologists according to the Istanbul Consensus and the Garden grading system. For cleavage-stage embryos, the cell number, homogeneity, and fragmentation rate, among other parameters, were determined and recorded. For blastocysts, the expansion state, development of the inner cell mass and trophoblast cells were evaluated, and blastocysts were graded according to the indicators.

### 2.6. Embryo transfer

Under the guidance of ultrasound monitoring, combined with the patient own conditions, 1 to 2 embryos were transferred to the patient uterus on the 3rd or 5th day after oocyte retrieval. If the fresh cycle was not suitable for transfer, the embryos (cleavage stage or blastocysts) were cryopreserved for subsequent frozen ET. Luteal support was routinely used after ET.

Serum hCG levels were measured 14 days after ET. If the results were positive, the patient was examined further for transvaginal ultrasonography 4 to 6 weeks after transfer to determine whether the gestational sac could be seen. Subsequent pregnancy results were obtained during follow-up at 6 weeks postpartum by contacting the patient by telephone.

### 2.7. Statistical methods

All data analyses were performed using SPSS 21.0 software. Continuous measurement data are expressed as the mean ± standard deviation (¯x ± s), and analysis of variance was used for comparisons between groups; discontinuous data are expressed as constituent ratios or rates (%), and the chi-square test was used for comparisons between groups. Multivariate logistic regression was used to adjust for confounding factors, and results are expressed as OR values and 95% CIs. The difference was considered to be statistically significant at *P* < .05.

## 3. Results

A total of 2377 first IVF/ICSI cycles that met the criteria from January 2015 to December 2020 were included in this study, and 1544 of them achieved at least one live birth, with a CLBR of 64.96%.

As shown in Table 1, there were significant differences in the baseline data of patients in different BMI groups. The age of Group A was 28.45 ± 5.32 years, the difference between the 3 groups was significant, and the proportion of tubal factor infertility was significantly higher in this group. In addition, compared with that in the other 2 groups of patients, the proportion of ovulatory disorders in the overweight and obese group was higher, accounting for 30.91%, and the proportion of primary infertility in Group C was also the highest among the 3 groups.

**Table 1:** Basic characteristics for patients with different BMI.

	Underweight	Normal	Overweight/obesity	*P*
	≤18.5 kg/m^2^ Group A	18.5–24.0 kg/m^2^ Group B	≥24.0 kg/m^2^ Group C	
No. of patients	250	1755	372	
Age (yr)	28.45 ± 5.32	29.89 ± 5.00	30.74 ± 5.40	.000
BMI (kg/m^2^)	17.67 ± 3.50	21.29 ± 3.29	26.45 ± 5.27	.000
Duration of infertility (yr)	3.88 ± 3.30	4.26 ± 3.36	4.70 ± 3.92	.000
Basic FSH (mIU/mL)	5.24 ± 3.25	5.75 ± 4.20	6.20 ± 4.25	.000
Antral follicle count	12.20 ± 6.30	12.07 ± 5.20	13.50 ± 5.01	.000
Type of infertility, n (%)				.000
Primary	118 (47.20)	870 (49.57)	190 (51.08)	
Secondary	132 (52.80)	885 (50.43)	182 (48.92)	
Main reason for infertility, n (%)				.000
Tubal factor	140 (56.00)	874 (49.80)	160 (43.01)	
Ovulation disorder	52 (20.80)	374 (21.31)	115 (30.91)	
Male factor	40 (16.00)	242 (13.79)	70 (18.82)	
Others	18 (7.20)	265 (15.10)	27 (7.26)	

Data were mean ± standard deviation unless otherwise noted.

BMI = body mass index, FSH = follicular-stimulation hormone.

The comparisons of pregnancy outcomes and CLBR among the 3 groups are shown in Table 2. Among them, the average number of oocytes retrieved in Group A was the highest at 11.25 ± 5.97, and the numbers of cleavage-stage and available D3 embryos were also the highest. Compared with that in the other 2 groups, the CLBR of patients in Group C was the lowest at 59.14%, and the difference was statistically significant.

**Table 2:** Treatment outcomes and cumulative live birth rate for patients with different BMI.

	Underweight	Normal	Overweight/obesity	*P*
	≤18.5 kg/m^2^ Group A	18.5–24.0 kg/m^2^ Group B	≥24.0 kg/m^2^ Group C	
No. of patients	250	1755	372	
No. of oocytes retrieved	11.25 ± 5.97	11.07 ± 5.49	10.52 ± 5.02	.000
No. of cleavage	9.08 ± 4.85	8.89 ± 4.27	8.20 ± 3.98	.000
No. of d 3 embryos	6.05 ± 4.21	5.82 ± 4.08	5.66 ± 3.64	.000
Cumulative live birth, n (%)	166 (66.40)	1158 (65.98)	220 (59.14)	.017

Data were mean ± standard deviation unless otherwise noted.

BMI = body mass index.

Based on the above results, we conducted a multivariate logistic regression analysis on the influencing factors of the CLBR of the young and older individuals (Table 3). Among younger patients, female BMI significantly affected the CLBR after adjustment for female age (aOR = 0.822, 95% CI: 0.817–0.957, *P* = .000). In the multivariate logistic analysis of the IVF/ICSI cycles of older patients, only female age was a risk factor affecting the CLBR, while the effect of overweight/obesity on the CLBR was not significant (aOR = 0.986, 95% CI: 0.903–1.027, *P* > .05).

**Table 3:** Factors associated with cumulative live birth rate in different age by multivariate logistic regression analysis.

	Young age	Advanced age
	<35 yr old (n = 1652)	≥35 yr old (n = 725)
	aOR (95% CI)	aOR (95% CI)
Age (yr)	0.801 (0.725–0.925)**	0.781 (0.703–0.895)**
BMI		
Underweight (≤18.5 kg/m^2^)	0.950 (0.922–1.204)	1.055 (0.966–1.128)
Normal (18.5–24.0 kg/m^2^)	1.000 (reference)	1.000 (reference)
Overweight/obesity (≥24.0 kg/m^2^)	0.822 (0.817–0.957)*	0.986 (0.903–1.027)

BMI = body mass index, aOR = adjusted odds ratio, CI = confidence interval.

* *P* < .05.

** *P* < .01.

## 4. Discussion

As one of the most basic methods of assisted reproductive technology, IVF has been widely used in the treatment of infertility, and the factors that affect the outcome of IVF pregnancy have always attracted the attention of clinicians and patients. In this study, the large sample data of our center were analyzed, and the CLBR was selected as the clinical outcome indicator for evaluating IVF/ICSI. The results of the study show that the age of the woman is still an important factor affecting the CLBR of IVF. In younger patients, overweight/obesity significantly affected the CLBR. However, in older patients, the effect of BMI on the CLBR was not significant.

In recent years, there has been much controversy regarding the impact of BMI on IVF pregnancy outcomes.^[9,10]^ Maintaining an appropriate BMI in women of childbearing age is very important for pregnancy and pregnancy maintenance. However, due to the accelerated pace of life now, irregular diets and endocrine disorders caused by various factors all lead to an increase in overweight or obesity. Being overweight or obese is not only a risk factor for infertility but also significantly reduces the clinical pregnancy rate and increases the risk of miscarriage among assisted reproduction technology patients.^[11]^ In 2020, a large-scale retrospective study found that whether underweight (<18.5 kg/m^2^), overweight (24–28 kg/m^2^) or obese (BMI > 28 kg/m^2^), the CLBR was significantly lower than that of normal body weight group, especially among younger patients, which is consistent with our findings.^[12]^ In addition, some studies have shown that in the process of IVF/ICSI treatment cycles, people with high BMI usually need more ovarian stimulation drugs, and some studies have found that the decidualization of endometrial stromal cells in obese women is impaired after pregnancy, the growth process of the early placenta is affected, and the rate of early miscarriage increases significantly.^[13]^ On the other hand, because being too thin is associated with the incidence of hypothalamic amenorrhea and anovulatory infertility,^[14,15]^ recent studies have also shown that compared with normal-weight women, underweight women have a lower cumulative pregnancy rate.

In previous studies, the relationship between female BMI and clinical pregnancy rate in IVF cycles has often been somewhat controversial.^[16,17]^ We speculate that the most important reason for the difficulty in unifying the conclusions is that there are many factors affecting the outcome of IVF outcomes, and there are obvious interactions.^[18]^ For example, there are significant differences in age among different BMI groups. In the population we included, in younger patients, the CLBR was significantly lower in the overweight/obese group. However, in the older population, BMI had no effect on the CLBR, indicating that in the older group, pregnancy outcomes were not sensitive to overweight/obesity.^[19]^ This also prompted us to carry out a large-sample cohort study, and it is necessary to analyze these confounding factors at the same time. In addition, we included only the first assisted pregnancy cycle to ensure the independence of each group of data, and the results were more objective.

In conclusion, the large-sample data of our center show that female age is still the main factor affecting the CLBR in the IVF/ICSI cycle. Female BMI did not affect the CLBR of older patients but significantly affected the CLBR of younger patients. Therefore, for young overweight/obese patients undergoing IVF/ICSI treatment, we still recommend active weight control before treatment to ensure better pregnancy outcomes.

## Author contributions

**Conceptualization:** Shuxia Ma.

**Data curation:** Shuxia Ma, Ruirui Li.

**Formal analysis:** Shuxia Ma, Lin Li.

**Funding acquisition:** Shuxia Ma.

**Supervision:** Shuxia Ma.

**Validation:** Lu Ouyang.
